# Development of the Experienced Communication in Dementia Questionnaire: A Qualitative Study

**DOI:** 10.1177/00469580211028181

**Published:** 2021-06-24

**Authors:** Maria W. L. J. Olthof-Nefkens, Els W. C. Derksen, Bert J. M. de Swart, Maria W. G. Nijhuis-van der Sanden, Johanna G. Kalf

**Affiliations:** 1 Zorggroep Maas & Waal, Beneden-Leeuwen, The Netherlands; 2 Radboud university medical center, Radboud Institute for Health Sciences, Department of Primary and Community Care, Nijmegen, The Netherlands; 3 Radboud university medical center, Donders Institute for Brain, Cognition and Behaviour, Department of Rehabilitation, Nijmegen, The Netherlands; 4 Radboud university medical center, Radboudumc Alzheimer Center, Nijmegen, The Netherlands; 5 Radboud university medical center, Radboud Institute for Health Sciences, Scientific Center for Quality of Care (IQ healthcare), Nijmegen, The Netherlands

**Keywords:** dementia, Alzheimer disease, neurodegenerative diseases, neurogenic communication disorders, qualitative research, surveys and questionnaires, speech-language pathology, language therapy

## Abstract

Communication problems with their caregivers are common in people with dementia. Although interventions for improvement of communication are being developed, a tool to measure how participants experience their communication is lacking. The objective of this article is to describe the development of a questionnaire that measures the “experienced communication” of persons with dementia (ECD-P) as well as of their caregivers (ECD-C). Interviews were conducted with five person with dementia—caregiver dyads who had recently received a new communication intervention. Reflexive thematic analysis was performed on the transcripts using ATLAS.ti. Codes were created, categories and themes were identified, and items for the questionnaires were generated. Selection of items and response scales was done in collaboration with the same dyads. The final version was established after pilot testing with seven other dyads and discussion with five experts in the field of dementia care. Analysis of the transcripts resulted in 212 codes and 17 categories within four themes: caregiver competence, social communication, communication difficulties in daily life, and experienced emotions during conversations. The final version of the ECD-P consists of part 1 with 22 items and 4-point Likert scales, and part 2 with two items and 1 to 10 scales. In the final ECD-C (proxy version), part 1 and part 2 are similar to the ECD-P, while a part 3 was added to assess caregivers’ own perspective and emotions (five items). Based on the experiences of people with dementia and their caregivers, we constructed a face-valid questionnaire. This justifies future research to test its clinimetric characteristics.

**What do we already know about this topic?**

Communication problems are common in people with dementia, but there is no tool that measures how persons with dementia and their caregivers experience their communication.

**How does your research contribute to the field?**

Together with the target group we developed a questionnaire that aims to measure the “experienced communication” of persons with dementia (ECD-P) as well as of their caregivers (ECD-C), that can be used to evaluate communication interventions.

**What are your research’s implications toward theory, practice, or policy?**

Although its clinimetric properties are not published yet, this face-valid list of items concerning the experienced communication of people with dementia and their caregivers is now available to healthcare professionals.

## Introduction

Dementia is a chronic condition that can be caused by a variety of neurodegenerative diseases. Alzheimer’s disease is the most prevalent cause of dementia, followed by vascular or multi-infarct dementia, frontotemporal degeneration, and Lewy Body dementia.^1^ Every type of dementia is dominated by cognitive decline, of which deterioration of language skills is an important symptom. These so-called “cognitive communication disorders” (CCDs) can arise in any phase of dementia and generally worsen during the course of the disease.^2^ CCDs cause misunderstanding, miscommunication, and emotional stress. They have a negative impact on personal relationships and daily activities^3,4^ not just for the person with dementia, but also for family, friends, and caregivers.^5,6^

Research on the quality and efficacy of communication between the person with dementia and informal caregiver is scarce.^7,8^ In neurodegenerative diseases, where cure is basically absent, there is a strong need for non-pharmacological interventions that alleviate symptoms and troublesome consequences.^9,10^ Currently, we are evaluating a short-term logopedic (intervention by a speech-language therapist (SLT)) intervention program for optimizing communication between people with dementia and their caregivers at the Radboudumc in Nijmegen, The Netherlands. This intervention does not aim to improve language skills (word finding, grammar, or comprehension), but seeks to enhance positive interaction (verbal and non-verbal) between the person with dementia and the caregiver. The main focus is 2-fold: on educating dyads about the influence of dementia on communication skills and on how to optimize these skills in a personalized manner, explicitly taking into account the narrative of the person with dementia.^11^ This intervention is expected to have a positive impact on how person with dementia-caregiver dyads experience their communication with each other and with the people in their social environment.^11^ However, an instrument to measure experienced communication was lacking. When searching the literature for valid instruments to measure this concept of “experienced communication,” we only retrieved generic or dementia-specific instruments that measure language performances^12-14^ or instruments that assess communication disorders on a functional level.^15,16^ These instruments are usually filled out by informal caregivers (proxy measures) only or based on observations by health care professionals, thereby neglecting valuable input from the persons with dementia themselves. Because we failed to find instruments that specifically measure experienced communication of people with dementia and their caregivers, we decided to create a new questionnaire, with one version for the person with dementia and one for the primary informal caregiver. Communication is a complex process between a sender and a receiver, where information is exchanged (verbal and non-verbal) and a continual switching of roles between senders and receivers takes place.^17^ Impaired communication skills disrupt this process on several levels, causing misunderstandings and frustration,^18^ and leading to stress, anxiety, and other negative feelings for both persons with dementia and caregivers.^5^ Since the aim of the logopedic intervention is to enhance positive interaction, and thereby diminish the negative consequences of CCDs, the questionnaire should contain items that correspond with the problems, feelings, and needs of persons with dementia and their caregivers. The aim of this article is to describe the development of this “Experienced Communication in Dementia” (ECD) questionnaire. The key research question was: what experiences did persons with dementia and their caregivers share about their communication difficulties and the impact of the intervention on these difficulties?

## Methods

To determine which aspects of daily communication should be reflected in the questionnaires, a qualitative study with elements of participatory research was conducted.

For the development of the ECD, we took the following steps^19^: (1) generating items from interviews, (2) selection of items and response scales, and (3) pilot testing the items. The execution of each step is explained in further detail in the following paragraphs.

### Participants

A purposive sampling strategy was applied. Person with dementia-caregiver dyads that had recently been treated with the new logopedic intervention^11^ at the Radboudumc in Nijmegen, the Netherlands, were invited to participate in a semi-structured, in-depth interview.

### Ethics

This study was approved by the regional medical ethics committee (file number 2014-1225). All participants were informed about the purpose and content of the study by researcher MO, both orally and in writing. All participants signed an informed consent form during their first meeting with researcher MO, knowing that their participation was voluntary and they had the right to withdraw at any time.

### Gathering Data from Interviews

The interviews were performed face-to-face by researcher MO, who is a speech language therapist with expertise in working with communicatively impaired elderly as well as a trained interviewer with interpersonal and communication skills (like openness, sensitivity, active listening, and reflecting), which are imperative when trying to elicit detailed information from participants.^20^ At the beginning of the interviews, aims and procedures were clearly explained. MO presented herself as a researcher, not mentioning her other role as an SLT, to avoid the suggestion of a therapeutic relationship. Additionally, she took ample time to establish a positive relationship, and if necessary she gave extra support to help persons with dementia remember and narrate their experiences, using cues like a photograph of their therapist or materials from the intervention. The interviews took place at the participants homes with both the person with dementia and the caregiver, also to make it possible for the caregivers to support the person with dementia and provide additional information if needed.^20^ A carefully constructed interview guide was used, containing open-ended questions about (A) the communication difficulties the dyads encountered (e.g. barriers and facilitators; experienced emotions; needs) and (B) the impact of the intervention on their lives (e.g. changes that occurred; experiences with given advices, exercises, and materials).

### Data Analysis

All interviews were audiotaped and transcribed verbatim. The transcripts were read, re-read and analyzed using ATLAS.ti by the first author and a research assistant. We applied reflexive thematic analysis, since this method best fits the study’s purpose for identifying patterns within data,^21^ in this case problems, emotions, and needs of participants regarding their communication difficulties. Thematic analysis encompasses an active role for the researcher in identifying themes and selecting which are of interest for the questionnaire.^21^ We followed the six recursive phases as described by Braun and Clarke^21^: familiarization by carefully reading the transcripts; an open coding cycle; generating initial categories and themes; reviewing and developing categories and themes; refining, defining and naming categories and themes; and writing up. This was an iterative process; analysis of a transcript was completed before conducting the next interview. This approach gave us the possibility to fine tune the interview questions and further specify the information given by the participants. Field notes and memos were created to provide insight in reasoning. The researchers conducted all coding processes independently, and discussed their findings after each coded transcript until consensus was reached on every code, every category, and every theme.

### Generating Items from Interviews

We looked for categories that described the problems, feelings, and needs of the participants, especially those that were also influenced by the logopedic intervention according to the participants. These categories were the starting point for formulating items that were deemed relevant for the assessment of experienced communication. We tried to stay as close as possible to the language used by the participants, as this is the language we wanted to use in the questionnaires. Therefore, we constantly switched back and forth between categories and quotes of participants. We created two versions of every item: one for the person with dementia and one for the caregiver. We intentionally kept the preliminary pool of items quite broad, to allow selection of the most suitable items for the final questionnaires.

### Selection of Items and Response Scales

In this phase of the study, we applied elements from participatory research by engaging in a collaborative partnership with the participants.^22^ We invited the same person with dementia-caregiver dyads to review all preliminary items and help us with the selection process. The dyads were visited for a second time at their homes by researcher MO. They were asked to reflect aloud on every item and the corresponding response scale. Then, researcher and dyads collaborated to make a selection of items for the questionnaires. Finally, these items were discussed within the research team (all authors of this article), and the first versions of the questionnaires were established.

### Pilot Testing the Questionnaires

To verify the relevance and comprehension of the questionnaires, the next step was pilot testing. First, the questionnaires were presented to several new person with dementia-caregiver dyads (on separate occasions) who had not received the logopedic intervention. They were recruited during their visit to the outpatient clinic of the Geriatrics Department of the Radboudumc in Nijmegen. They were asked to articulate their thoughts while responding to all items (the “think aloud technique”).^23^ Notes were kept during this process. Second, the questionnaires were discussed with experts in the field of dementia or communication disorders from the Radboud Alzheimer Center. All comments were used to make final adjustments in wording and sequence of the items. Then, the research team (also the authors of this article) decided on the final versions of the questionnaires.

## Results

### Participants

Five person with dementia-caregiver dyads could be invited for an interview, and all of them agreed to participate. Their characteristics are shown in Table 1. Except from the daughter in law, all participants were retired from work.

**Table 1.** Participant Characteristics.

Dyad	Sex PwD	Age PwD	Diagnosis	Disease duration*	Sex caregiver	Relationship
1	Man	80 years	Vascular dementia	5 years	Woman	Spouse
2	Man	66 years	Alzheimer’s disease	4 years	Woman	Spouse
3	Man	75 years	Alzheimer’s disease	2 years	Woman	Daughter in law
4	Man	59 years	Primary progressive aphasia	3 years	Woman	Spouse
5	Man	76 years	Primary progressive aphasia	9 years	Woman	Spouse

PwD = person with dementia.

* Time since diagnosis, not since first symptoms.

### Generating Items from Interviews

The interviews with the dyads lasted between 45 and 75 minutes. The open coding process of the complete transcripts resulted in 212 codes. We generated 17 relevant categories which we classified within four themes. An overview of the four themes (**bold**) and categories is shown in Table 2, and illustrated with a quote for every category from a person with dementia (PwD) or caregiver (CG).

**Table 2.** Themes, Categories, and Quotes from Interviews.

Themes	Categories	Example quotes
**Caregiver competence**	*Adaptation of speech and language (by the caregiver)*	“You [as a caregiver] have to be very aware of what you say, how to say it, where to say it, and so on.” (CG)
		“When I am in the kitchen, and I ask him [the PwD] something, he does not respond anymore. I need to go to him and make eye contact.” (CG)
	*Need of information about dementia in relation to communication*	“This situation, it is all new to us. Things happen because of the dementia. That is what I have missed; practical advices on how to deal with these things.” (CG)
		“We just wanted to know: what is happening, and what is the prognosis? But we did not get a prognosis, they just don’t tell you what to expect.” (CG)
**Social communication**	*Experiences during group meetings*	“He [the PwD] is bothered by the presence of more people. The moment the group gets bigger, he shuts down completely and nothing comes out anymore. A conversation with one other person still works fairly well, but in groups [. . .] it is just not possible anymore.” (CG)
		“He [the PwD] withdraws from conversations more and more.” (CG)
	*Interests and social activities*	“I’m not going anymore [to a monthly meeting with like-minded people], I don’t like it anymore.” (PwD)
	*Openness about the disease*	“He [the PwD] did not want to tell anyone about his disease. [. . .] But eventually we told our children. He found that very hard, but he also noticed that it gave him some peace of mind. He did not have to pretend that he was doing fine anymore.” (CG)
		“I am very clear to everyone [about having Alzheimer’s disease], from the beginning, I have always done that. Then you get good other things.” (PwD)
	*Reactions of others to communication difficulties*	“Due to the communication difficulties, family and friends visit less often than they did in the past. They feel insecure about how to approach him [the PwD].” (CG)
**Communication difficulties in daily life**	*Barriers for communication*	“When he [the PwD] gets nervous or stressed, communication gets more difficult. How well it goes highly depends on the setting he is in.” (CG)
	*Facilitators for communication*	“I notice that the people you [the PwD] can easily talk to, are usually the people that are patient and really listen to you. People who give you the feeling that they understand what’s on your mind.” (CG)
		“One on one conversations in a quiet environment tend to be going the best.” (CG)
	*Person with dementia has problems with language comprehension*	“He [the PwD] does not understand when people speak too fast.” (CG)
		“There are more misunderstandings between us because of the communication difficulties.” (CG)
	Person with dementia does not make telephone calls anymore	“When the phone rings, he [the PwD] does not answer anymore. [. . .] Questions of the person who calls require a fast response, and that is too difficult for him.” (CG)
	Person with dementia experiences reading difficulties	“He [the PwD] can no longer read the newspaper.” (CG)
	Person with dementia experiences speaking difficulties	“He [the PwD] has better days and worse days. Then it seems like he almost cannot speak anymore.” (CG)
		“I used to talk fast, and I still want to. But I can’t anymore.” (PwD)
	Person with dementia has trouble watching television	“He could no longer read the subtitles, so for a long time I read them out loud so he could still understand what the program was about.” (CG)
	*Word finding difficulties*	“He [the PwD] talks in telegram style, and he often uses the wrong words.” (CG)
	Person with dementia experiences writing difficulties	“Writing is not possible anymore.” (CG)
**Experienced emotions of PwD**	*Emotions of person with dementia when anticipating conversations*	“He talks less and less, and becomes very nervous and anxious when he knows that he has to talk to people.” (CG)
	*Emotions of person with dementia when communication breaks down*	“He [the PwD] is sometimes difficult to handle, when he gets angry with himself because of the communication difficulties.” (CG)
		“I feel very sad [when communication breaks down]. It is not very dramatic or so, but I feel uncomfortable when it happens.” (PwD)

The 12 categories in *italic* were described by the participants as problems, feelings, or needs with regards to communication difficulties (part A of the interview guide), as well as being subject to change by the intervention (part B of the interview guide). In the construction of the items, our aim was to stay as close as possible to the language in the quotes of the interviewees. For example, the category “Reactions of the person with dementia to communication difficulties” consists of two items: “I try to avoid events were there are many people present” and “I continue to participate in conversations, although I find it difficult to do so.” Eventually two preliminary pools of 43 items were composed: one pool for the person with dementia and one with comparable items from the perspective of the caregiver. An example for this change of perspective: “I’ve become more quiet than I used to be” for the person with dementia and “My partner has become more quiet than he/she used to be” for the caregiver. We used the word “partner” to refer to the person with dementia, but in the instructions of the questionnaires it is explained that for “partner” also mother, father, or any other relation can be read.

Finally, a suitable response scale was assigned to every item. The first scale for satisfaction contained five colored smiley’s ranging from happy (green) to sad (red) (17 items). The second scale for frequency had the following response options: never, monthly, weekly, daily, in every conversation (7 items). The third scale for agreement contained the following five response options: fully disagree, partially disagree, neutral, partially agree, fully agree (17 items). The fourth scale was a grading between 1 (poor) and 10 (excellent) for the quality of conversations (2 items).

### Selection of Items and Response Scales

All 43 items were field-tested and discussed extensively with the same five persons with dementia-caregiver dyads. Based on their experiences and input, 19 items were eliminated that either were too abstract, too difficult to respond to, or too specific. Some examples of these items: “My caregiver arranges for a quiet environment when we talk to each other” was too difficult to respond to by the persons with dementia. The item “People in my social environment give me enough time to react during a conversation” was not applicable to all participants and therefore considered not adequate enough to be in the questionnaires.

Although we presumed that the colored smiley response options would be helpful, all persons with dementia told us that they disliked the smileys or did not fully understand their meaning. In collaboration with these five dyads, the research team decided to delete the neutral response option, thus changing the 5-point Likert scales for frequency and agreement to 4-point scales. This was done because everyone with communication difficulties is supposed to have an opinion about these topics, while a neutral response is meaningless.

Finally, five items were added about the caregiver’s personal perspective and emotions. According to the caregivers, their emotions (eg, sadness, anger, and frustration) obviously have an impact on the interaction with the person with dementia, and these emotions had also changed during and after the intervention. Therefore these items were added as a separate part of the caregiver version.

### Pilot Testing the Questionnaires

In a first round of the pilot testing of the questionnaires we consulted seven dyads (whereof three women and four men with early stage dementia). In a second round the items were discussed by five experienced health care professionals: a geriatrician, a physician assistant, and three SLTs. This phase led to some changes in wording to improve comprehensibility. Two examples of sentences that were adapted: “I use words that are wrong” was changed into “I can’t find the right words,” and “I withdraw from conversations” was changed into “I tend to withdraw from conversations.” Lastly, the sequence of the items was discussed with all dyads and health care professionals. We decided to bundle items from the same themes, since this would prevent persons with dementia from having to make too many topic shifts. After these adjustments, the final versions of both questionnaires were established.

**Figure 1.**
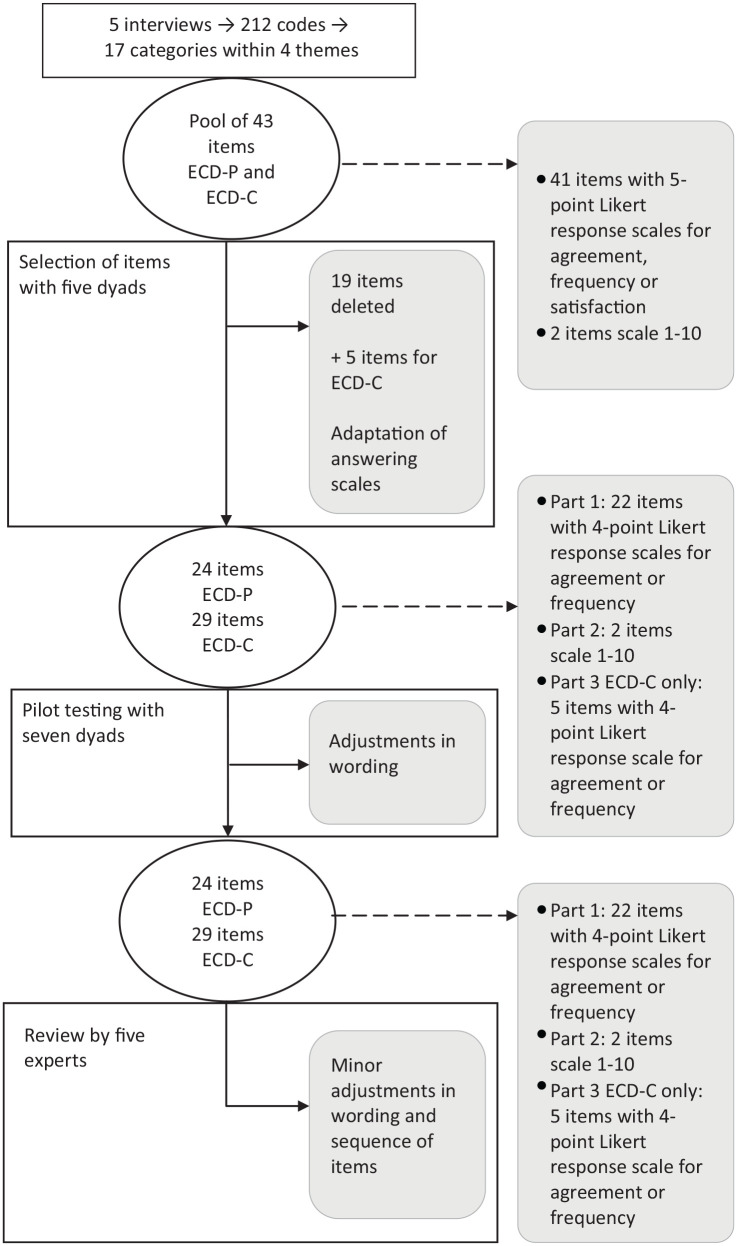
Flow chart of the development process of the ECD questionnaire. ECD-P = version for the person with dementia; ECD-C = caregiver version.

The development process of the questionnaires is also shown in the flow chart (Figure 1).

### Final Result: ECD-P and ECD-C

The items and corresponding score options (3 = strongly disagree to 0 = strongly agree) of the final ECD-P and ECD-C are displayed in Tables 3 and 4. For four items we reversed the score options (0 = strongly disagree to 3 = strongly agree), because we wanted to stay close to the words that had been used by the participants. The items were translated into English by the first author for the purpose of this article only.

**Table 3.** Experienced Communication in Dementia Questionnaire—Version for the Person with Dementia.

Parts	Themes	Items	Response options	Scores	
Part 1	Caregiver competence	1. My caregiver makes an effort to understand me	Strongly disagree—disagree—agree—strongly agree	3—2—1—0	Min. score: 0 Max. score: 66
		2. My caregiver usually talks at a pleasant pace (not too fast and not too slow)	Strongly disagree—disagree—agree—strongly agree	3—2—1—0	
		3. My caregiver makes eye contact when we talk to each other	Strongly disagree—disagree—agree—strongly agree	3—2—1—0	
		4. I feel safe in conversations where my caregiver is present	Strongly disagree—disagree—agree—strongly agree	3—2—1—0	
		5. My caregiver and I talk less and less to each other	Strongly disagree—disagree—agree—strongly agree	0—1—2—3	
	Social communication	6. I’ve become more quiet than I used to be	Strongly disagree—disagree—agree—strongly agree	0—1—2—3	
		7. I tend to withdraw from conversations	Strongly disagree—disagree—agree—strongly agree	0—1—2—3	
		8. I try to avoid events where there are many people present	Strongly disagree—disagree—agree—strongly agree	0—1—2—3	
		9. I like to be helped when I experience communication breakdown	Strongly disagree—disagree—agree—strongly agree	3—2—1—0	
		10. I tell people when I get stuck in a conversation	Strongly disagree—disagree—agree—strongly agree	3—2—1—0	
		11. I tell people about my illness	Strongly disagree—disagree—agree—strongly agree	3—2—1—0	
		12. People in my social environment adjust to my communication problems	Strongly disagree—disagree—agree—strongly agree	3—2—1—0	
		13. I am satisfied with my current social contacts	Strongly disagree—disagree—agree—strongly agree	3—2—1—0	
		14. Friends and acquaintances come to visit as often as they did in the past	Strongly disagree—disagree—agree—strongly agree	3—2—1—0	
	Communication difficulties in daily life	15. I can’t find the right words	During every conversation—every day—every week—(almost) never	3—2—1—0	
		16. I am not able to participate because the conversation goes too fast	During every conversation—every day—every week—(almost) never	3—2—1—0	
		17. There are misunderstandings between me and my caregiver	During every conversation—every day—every week—(almost) never	3—2—1—0	
	Experienced emotions of PwD	18. I feel nervous during a conversation	During every conversation—every day—every week—(almost) never	3—2—1—0	
		19. I feel frustrated during a conversation	During every conversation—every day—every week—(almost) never	3—2—1—0	
		20. I feel sad during a conversation	During every conversation—every day—every week—(almost) never	3—2—1—0	
		21. I feel angry during a conversation	During every conversation—every day—every week—(almost) never	3—2—1—0	
		22. I feel anxious during a conversation	During every conversation—every day—every week—(almost) never	3—2—1—0	
Part 2	Assessment of conversation quality	23. In general, I would grade the conversations between me and my partner with an:	(poor) 1—2—3—4—5—6—7—8—9—10 (excellent)		Min. score: 2 Max. score: 20
		24. In general, I would grade the conversations between me and the people in our immediate surroundings (children, friends, neighbors, etc.) with an:	(poor) 1—2—3—4—5—6—7—8—9—10 (excellent)		

**Table 4.** Experienced Communication in Dementia Questionnaire—Caregiver Version.

Parts	Themes	Items	Response options	Scores	
Part 1	Caregiver competence	1. I make an effort to understand my partner	Strongly disagree—disagree—agree—strongly agree	3—2—1—0	Min. score: 0 Max. score: 66
		2. I usually talk at a pleasant pace (not too fast and not too slow)	Strongly disagree—disagree—agree—strongly agree	3—2—1—0	
		3. I make eye contact with my partner when we talk to each other	Strongly disagree—disagree—agree—strongly agree	3—2—1—0	
		4. My partner feels safe in conversations where I am present	Strongly disagree—disagree—agree—strongly agree	3—2—1—0	
		5. My partner and I talk less and less to each other	Strongly disagree—disagree—agree—strongly agree	0—1—2—3	
	Social communication	6. My partner has become more quiet than he/she used to be	Strongly disagree—disagree—agree—strongly agree	0—1—2—3	
		7. My partner tends to withdraw from conversations	Strongly disagree—disagree—agree—strongly agree	0—1—2—3	
		8. My partner tries to avoid events where there are many people present	Strongly disagree—disagree—agree—strongly agree	0—1—2—3	
		9. My partner likes to be helped when he/she experiences communication breakdown	Strongly disagree—disagree—agree—strongly agree	3—2—1—0	
		10. My partner tells people when he/she gets stuck in a conversation	Strongly disagree—disagree—agree—strongly agree	3—2—1—0	
		11. My partner tells people about his/her illness	Strongly disagree—disagree—agree—strongly agree	3—2—1—0	
		12. People in our environment adjust to my partner’s communication problems	Strongly disagree—disagree—agree—strongly agree	3—2—1—0	
		13. My partner is satisfied with his/her current social contacts	Strongly disagree—disagree—agree—strongly agree	3—2—1—0	
		14. Friends and acquaintances come to visit as often as they did in the past	Strongly disagree—disagree—agree—strongly agree	3—2—1—0	
	Communication difficulties in daily life	15. My partner can’t find the right words	During every conversation—every day—every week—(almost) never	3—2—1—0	
		16. My partner is not able to participate because the conversation goes too fast	During every conversation—every day—every week—(almost) never	3—2—1—0	
		17. There are misunderstandings between me and my partner	During every conversation—every day—every week—(almost) never	3—2—1—0	
	Experienced emotions of PwD	18. My partner feels nervous during a conversation	During every conversation—every day—every week—(almost) never	3—2—1—0	
		19. My partner feels frustrated during a conversation	During every conversation—every day—every week—(almost) never	3—2—1—0	
		20. My partner feels sad during a conversation	During every conversation—every day—every week—(almost) never	3—2—1—0	
		21. My partner feels angry during a conversation	During every conversation—every day—every week—(almost) never	3—2—1—0	
		22. My partner feels anxious during a conversation	During every conversation—every day—every week—(almost) never	3—2—1—0	
Part 2	Assessment of conversation quality	23. In general, I would grade the conversations between me and my partner with an:	(poor) 1—2—3—4—5—6—7—8—9—10 (excellent)		Min. score: 2 Max. score: 20
		24. In general, I would grade the conversations between my partner and the people in our immediate surroundings (children, friends, neighbors, etc.) with an:	(poor) 1—2—3—4—5—6—7—8—9—10 (excellent)		
Part 3	Communication difficulties in daily life	25. I find it tiring to interact with my partner	Strongly disagree—disagree—agree—strongly agree	0—1—2—3	Min. score: 0 Max. score: 21
		26. It burdens me that communication is becoming increasingly difficult	Strongly disagree—disagree—agree—strongly agree	0—1—2—3	
	Experienced emotions of caregiver	27. I feel angry during a conversation	During every conversation—every day—every week—(almost) never	3—2—1—0	
		28. I feel sad during a conversation	During every conversation—every day—every week—(almost) never	3—2—1—0	
		29. I feel frustrated during a conversation	During every conversation—every day—every week—(almost) never	3—2—1—0	

The ECD-P consists of two parts with a total of 24 items: one part with 22 items and one part with 2 items. The ECD-C is comparable, but items are formulated from the perspective of the caregiver and it contains a third part of 5 items (total 29 items). We consider part 1 of both questionnaires as “the body” of the instrument, because these parts contain items about all four themes: caregiver competence, social communication, communication difficulties in daily life and experienced emotions during a conversation.

Response options are 4-point Likert scales, either for agreement (“fully disagree-partially disagree-partially agree-fully agree”) or for frequency (during every conversation-every day-every week-(almost) never). Parts 2 of both versions contain two items for assessment of conversation quality between the person with dementia and the caregiver and between the person with dementia and closest family members and friends. These items are to be answered on a scale of 1 (poor) to 10 (excellent). Part 3 is for ECD-C only, and contains five items regarding the caregivers’ own perspective and emotions about the current situation, with the same 4-point response scales as part 1 (agreement or frequency).

Finally, scores between 0 and 3 were assigned to every response option. A lower score is an indication for a more positive experienced communication, a higher score indicates a more negative experienced communication.

## Discussion

This qualitative study resulted in a carefully constructed and face-valid new tool aimed to grasp changes in the experienced communication of a person with dementia and his or her caregiver. In the following paragraphs we will discuss this result and the next steps to the validation and implementation of the ECD.

Involving persons with dementia and their caregivers in the development of the ECD was an inspiring experience, and empowering for both researchers and dyads. The persons with dementia were capable to remember and verbalize their experiences, even though this required patience and sometimes visual cues or verbal repetition of the last sentences by the researcher or assistance of the caregivers. Their valuable input was endorsed in the phase where we selected the items and response scales together with the dyads: the recognition and acknowledgement of the items by the participants confirmed that we had distilled appropriate information from the initial interviews and used adequate wording in the questionnaire. Based on our experiences, we suspect that self-administration of the ECD by persons with dementia could be difficult, depending on the severity of the dementia as well as on the level of literacy. We therefore recommend always administering the ECD in the presence of a speech and language therapist (SLT), researcher, or other trained professional, who can also conduct the questionnaire as an interview if this is preferred. Joint interviews are commonly used in quality of life research and have already proven to be a reliable method to assess characteristics of people with dementia.^24,25^

During this study, it became clear that the participants described a wide range of problems, feelings, and needs related to communication difficulties, which is inherent to the complexity of communication and the many factors that are involved.^26^ Topics that were highly prevalent and relevant to one dyad could be far less of an issue to another. Our aim was to capture this variation, which meant that we had to be very considerate about the wording of the items, as well as the response options. By involving persons with dementia, caregivers and health care professionals in every step of the development process we hope to have optimized the likelihood that the ECD questionnaires are acceptable to future users.

Previous research by Muò et al^27^ provided a detailed description of dementia-associated disabilities in people with Alzheimer’s disease through the International Classification of Functioning, Disability and Health (ICF) model for classification of human functioning.^28^ We noticed that the four themes that we extracted from the interview transcripts, and the fifth of “caregiver emotions” correspond with significant components in the ICF model (activities, participation, personal, and environmental factors). This suggests that our study has resulted in a valid coverage of relevant aspects of communication in dementia.

We observed that administration of the ECD is feasible within a limited timeframe (less than 15 minutes, but this will be tested more accurately in a future study), which is helpful for application in clinical practice. Objective assessments are usually much more time consuming, for instance the recently developed “Verbal and Nonverbal Interaction Scale for Care Recipient” (VNVIS-CR).^29^ This is a reliable and valid observer rating scale that provides relevant information about verbal and nonverbal communication skills of persons with dementia. Application of this scale requires multiple video-recorded conversations from daily situations at participants homes, which have to be analyzed by researchers or other trained professionals, taking considerable amounts of time. Adequate evaluation requires both objective and subjective measurements and comparison of our new tool with a clinician-rated instrument like the VNVIS-CR seems a relevant next step, even though it measures a different but related concept.

Although the construction of twin-questionnaires—a patient measure and a comparable proxy measure—is relatively new to the field of speech and language therapy, it has been long used in dementia care research with quality of life questionnaires.^24,30,31^ Logsdon et al^30^ describe that reports from persons with dementia and caregivers are related, but not identical. It is also found that people with dementia tend to give higher rates to their quality of life than their caregivers do.^30,31^ We suspect this phenomenon might also occur in ECD scores, so it is important to keep this in mind when interpreting, comparing, and discussing ECD scores. Finally, previous research has also shown that even moderate levels of cognitive impairment did not have a negative impact on reliability or validity of the outcomes.^27^ We therefore consider the use of the ECD to be enriching for both research on communication interventions as well as for clinical practice by SLTs. Information from both conversation partners can support SLTs to identify individualized therapy goals or areas that need specific attention during therapy sessions, and to address differences in experiences between persons with dementia and caregivers.

A potential limitation of this study is that we based the items of the questionnaires on five interviews, and that all persons with dementia were men. This was due to the small number of people who already had received the communication intervention not too long before the interviews were conducted. Caregiver management strategies differ between women and men, and are important predictors for patient agitation and caregiver burden.^32^ These strategies might also affect communication skills of both persons involved, which potentially could have influenced our results. It was however a considered choice to recruit only persons who underwent our logopedic intervention, since they acknowledged their communication difficulties and they could also reflect on which elements of the intervention were helpful for them. The pilot-testing round showed us that seven new dyads (whereof both male and female persons with dementia), who had not received the logopedic intervention, also recognized and related to the items we formulated. Additionally, the last transcript that was coded, did not elicit new and relevant information compared to the four previous transcripts. However, we recommend that future research should include a broader sample with better distribution of participants demographics.

We included people with various types of dementia and disease durations, which we consider to be a potential limitation as well as a strength. It helped us to cover a broad range of topics and making the ECD items as recognizable as possible to future users. On the other hand, as dementia progresses, language skills deteriorate in each type.^2^ This made the interviews challenging sometimes, especially with participant 5, who had been affected by Primary Progressive Aphasia for 9 years. But as described above, with patience and (visual) help from the researcher and caregiver, and continuously checking whether we understood him correctly, this person also contributed to the development of the ECD in a meaningful way.

The ECD now seems face-valid, but its usefulness needs to be established by clinimetric research to test reproducibility and validity and also its ability to grasp improvement or deterioration of experienced communication. Until then, a list of items is available that has carefully put the experienced communication of people with dementia and their caregivers into words.
